# Poster Session II - A307 BEYOND THE USUAL SUSPECT: RARE COEXISTENCE OF CROHN’S DISEASE AND COLLAGENOUS COLITIS

**DOI:** 10.1093/jcag/gwaf042.307

**Published:** 2026-02-13

**Authors:** A Samadi, J Baratz, M Cino, P Tandon, A N Sasson

**Affiliations:** Toronto Western Hospital, Toronto, ON, Canada; Toronto Western Hospital, Toronto, ON, Canada; Toronto Western Hospital, Toronto, ON, Canada; Toronto Western Hospital, Toronto, ON, Canada; Toronto Western Hospital, Toronto, ON, Canada

## Abstract

**Background:**

Inflammatory bowel disease (IBD) is a chronic intestinal inflammatory condition made up of mainly Crohn’s disease and ulcerative colitis. Microscopic colitis (MC) is another distinct form of colitis which is subdivided to lymphocytic and collagenous colitis. While IBD and MC share several pathophysiologic mechanisms, they differ in both endoscopic and histologic findings, along with their method of treatment and management. The co-occurrence of MC in patients with underlying IBD is rare and sparsely reported in literature.

**Aims:**

We present a unique case of a patient with a diagnosis of Crohn`s disease (CD) on advanced therapy with a change in both clinical pattern and histopathology to support a diagnosis of MC, with a collagenous variant. We also systematically reviewed the current literature on this atypical finding.

**Methods:**

A search of electronic databases was conducted up to September 2025 for all studies and reviews involving patients with a dual diagnosis of IBD and MC.

**Results:**

A 47-year-old woman initially presented with a history of diarrhea and abdominal pain beginning at age 18. Due to persistent symptoms, she underwent a colonoscopy with endoscopic and histologic findings supporting a diagnosis of non-stricturing, non-penetrating ileocolonic Crohn’s disease. She was placed on 5-aminosalicylic acid (5-ASA) therapy which was eventually escalated to an advanced biologic therapy, infliximab. Her disease had overall remained quiescent though she experienced mild flares with histology on surveillance describing moderate acute colitis with cryptitis, crypt abscesses, and atrophic glands, supporting Crohn’s disease. One year later, she experienced a recurrent flare-up, though this time pathology revealed lymphoplasmacytic inflammation with cryptitis and crypt epithelial apoptosis, interpreted as drug-induced colitis. Her symptoms continued and she was started on a trial of budesonide. Another year later, following the discontinuation of budesonide, her diarrhea recurred. She underwent colonoscopy for reassessment, which was grossly normal; however, random biopsies demonstrated collagenous colitis with intraepithelial lymphocytosis as well as thickening of the collagen plate, highlighted by Masson’s trichrome. Her concurrent diagnosis of MC had been challenging to manage with ongoing need for budesonide therapy and persistent histology supporting collagenous colitis. As such, her therapeutic regimen was adjusted to a different biologic agent, vedolizumab, and clinical remission was achieved.

**Conclusions:**

Physicians should consider MC as a differential diagnosis in patients who develop diarrhea despite quiescent colitis, particularly when colonoscopy findings are grossly normal. Accurate diagnosis can help early management of the disease and prevent unnecessary escalation of IBD therapy, unless otherwise clinically indicated.

**Funding Agencies:**

None

